# Effects of early postnatal environment on hypothalamic gene expression in OLETF rats

**DOI:** 10.1371/journal.pone.0178428

**Published:** 2017-06-02

**Authors:** Yonwook J. Kim, Mariana Schroeder, Nu-Chu Liang, Timothy H. Moran, Aron Weller, Sheng Bi

**Affiliations:** 1 Department of Psychiatry and Behavioral Sciences, Johns Hopkins University School of Medicine, Baltimore, Maryland, United States of America; 2 Psychology Department and Gonda Brain Research Center, Bar Ilan University, Ramat-Gan, Israel; Western University of Health Sciences, UNITED STATES

## Abstract

Previous reports have shown that the early postnatal environment has the ability to modify the obesity phenotype of Otsuka Long-Evans Tokushima Fatty (OLETF) rats. To determine whether this early postnatal environment affects hypothalamic signaling systems involved in energy balance, OLETF pups and lean Long-Evans Tokushima Otsuka (LETO) pups were cross-fostered to same or opposite strain Dams (designated as LdLp: LETO pups with LETO dams; LdOp: OLETF pups with LETO dams; OdLp: LETO pups with OLETF dams; and OdOp: OLETF pups with OLETF dams). Hypothalamic gene expression was examined at postnatal day 23 (PND 23) and PND 90 as OdOp rats started to gain more body weight at PND 23 and developed obesity at PND 90 relative to lean control LdLp rats. On PND 23, neuropeptide Y (*Npy*) gene expression was significantly increased in the dorsomedial hypothalamus (DMH) in both LdOp and OdOp pups compared to LdLp pups. Maternal environment did not affect DMH *Npy* expression in LETO weanlings. On PND 90, maternal environment during the cross-fostering period had a major effect on DMH *Npy* expression. Levels were significantly increased in both OdOp and OdLp rats relative to those in LdOp rats and LdLp controls. Reduced expression of *Npy* in the DMH of LdOp rats was consistent with their reduction of body weight compared to OdOp rats. In contrast to DMH *Npy*, gene expression for *Npy* and proopiomelanocortin in the arcuate nucleus appeared to appropriately respond to alterations in body weight and plasma leptin levels. Levels of oxytocin gene expression in the paraventricular nucleus were lower in offspring raised by LETO dams apparently responding to the higher DMH NPY levels. Together, our results demonstrate effects of both genotype and early postnatal environment on obesity of OLETF rats and further suggest an important role of DMH NPY in the development of obesity of OLETF rats.

## Introduction

Recent evidence has indicated the importance of maternal environment during pregnancy and lactation in determining the programming of later sensitivity or resistance to obesity in the offspring. Both low birth weight and increased birth weight and/or adiposity at birth become a high-risk factor for child/adult glucose intolerance, diabetes and obesity [1–4]. Similar results have been demonstrated in studies in rodents [5,6]. Animal studies have also shown that cross fostering has the ability to modulate the metabolic phenotype of offspring, attenuating the obesity of obesity prone mice, and inducing obesity and insulin resistance in normally obesity-resistant mice [7]. Moreover, the postnatal environment overcomes both genetic predisposition and prenatal factors in determining the development of adiposity, insulin sensitivity, and the brain pathways that mediate these functions in obesity-prone rats [8]. The postnatal environment also attenuates leptin resistance in the offspring of dams fed a high fat diet during pregnancy [9]. However, much of the work that has characterized the effects of postnatal environment has used polygenic rodent models of obesity and it is not clear how the postnatal maternal environment may modulate specific neural signaling in models with an identified genetic defect.

The Otsuka Long–Evans Tokushima Fatty (OLETF) rat is an outbred animal model of obesity and non-insulin-dependent diabetes mellitus (NIDDM) that has been characterized as having features of hyperphagia (in both dark and light periods), mild obesity, late onset of hyperglycemia, hyperinsulinemia and hyperleptinemia [10,11]. Genetic analysis has revealed a congenital defect in the expression of the cholecystokinin (CCK) 1 receptor gene in this animal model [12]. Within the hypothalamus, CCK-1 receptors and neuropeptide Y (NPY) are co-localized in dorsomedial hypothalamic (DMH) neurons [13]. We have proposed an etiological role for NPY in the DMH in the development of hyperphagia and obesity of OLETF rats due to a deficit in the control of DMH NPY in these animals. *Npy* gene expression is significantly elevated in the DMH of pair-fed OLETF rats that are given the amount of food consumed by lean Long-Evans Tokushima Otsuka (LETO) rats [14]. This NPY overexpression is also evident in the DMH of pre-obese young OLETF rats [14,15]. Moreover, knockdown of NPY in the DMH ameliorates the hyperphagia, obesity and impaired glucose tolerance of OLETF rats [16], whereas viral-mediated overexpression of NPY in the DMH of intact rats causes hyperphagia and obesity [17]. Recently, we have categorized the maternal environmental contribution to adult sensitivity and resistance to obesity in OLETF rats, demonstrating a moderating role for the postnatal maternal environment on the obesity trajectory and food intake of OLETF rats [18,19]. In this study, we aimed to determine whether maternal environment alters *Npy* expression in the DMH, and in this way, affects the development of obesity of OLETF rats. Within the hypothalamus, the anorexigenic peptides pro-opiomelanocortin (POMC, a precursor of alpha-melanocyte stimulating hormone), corticotropin-releasing factor (CRF) and oxytocin (OXY) play an important role in the control of food intake and energy balance, and dysregulation of these peptides contributes to the obesity. Thus, we also examined hypothalamic expression of *Pomc*, *Crf* and *Oxy* in the present study.

## Materials and methods

### Cross fostering

OLETF and LETO rats were raised in the colony of the Gonda Brain Research Center at Bar-Ilan University, Ramat-Gan, Israel. The founder rats were received as a generous gift from the Tokushima Research Institute, Japan. OLETF and LETO offspring were housed together with their dams and litters until weaning and in unisex pairs from then on. Rats were housed in polycarbonate cages (23.5 cm height 26.5 cm width 43 cm length), with stainless steel wire lids and wood shavings as the bedding material. Standard chow (2018S Teklad Global, 5% fat) and water were always available. The animals were maintained on a 12:12 hr light:dark cycle, with lights on at 06:00. The pregnant females were checked daily for parturition. Newborn litters found until 12:00 hr each day were designated as born on that day (PND 0). On PND 1, litters were culled to 10 pups when a large litter was born. Litters with less than 8 pups were excluded from the experiment. Sex distribution was kept as equal as possible in each litter. At the time of culling, entire litters were fostered to another dam, either from the same or the opposite strain. The groups were labeled as follows: the LETO pups fostered to LETO dam were designated LdLp (LETO dam—LETO pups); the OLETF pups fostered to OLETF were designated OdOp (OLETF dam -OLETF pups); the LETO pups fostered to OLETF dams were designated OdLp (OLETF dam–LETO pups); the OLETF pups fostered to LETO were designated LdOp (LETO dam–OLETF pups). Six to seven litters were used per group. Data represent 1 pup per litter. All pups were weaned to standard chow (PND 22). The research protocol was approved by the Institutional Animal Care and Use Committee of Bar-Ilan University.

### Body weight

Body weights of the rats were measured every fifth day from birth to sacrifice day (PND 23 or PND 90).

### Tissue collection

Male rats were sacrificed at two time points: at weaning (PND 23) and at PND 90. On the sacrifice day, rats were weighed and sacrificed between 11:00 AM and 2:00 PM as previously reported [15,19]. After decapitation, brains were removed and rapidly frozen for subsequent determinations of hypothalamic *Npy*, *Pomc*, *Crf* and *Oxy* mRNA expression using in situ hybridization. All samples were preserved at −80°C until analyzed.

### In situ hybridization

Coronal sections were cut at 14 μm via a cryostat, mounted on superfrost/plus slides (Fisher Scientific, Fair Lawn, NJ) in a 1:6 series, fixed with 4% paraformaldehyde, and stored at -80°C for *in situ* hybridization determination. Sections at levels over the compact subregions of the DMH were taken for determination of arcuate nucleus (ARC) *Npy*, ARC *Pomc*, and DMH *Npy* mRNA expression levels [15] and sections at the level of the paraventricular nucleus (PVN) were taken for determination of PVN *Crf* and *Oxy* mRNA levels. One slide per series was stained with cresyl violet acetate to allow sections to be anatomically matched among animals.

^35^S-labed *Npy*, *Pomc*, *Crf* and *Oxy* antisense riboprobes were transcribed from rat *Npy* precursor cDNA, mouse full-length *Pomc* cDNA, rat *Crf* precursor cDNA, or rat *Oxy* cDNA as previously described [14,20] by using in vitro transcription systems (Promega, Madison WI) and purified by Quick Spin RNA columns (Roche, Indianapolis, IN). The fixed, frozen tissues sections were allowed to warm to room temperature, treated with acetic anhydride, dehydrated in gradient alcohol, air-dried, and incubated in hybridization buffer containing 50% formamide, 0.3 M NaCl, 10 mM Tris/Cl (pH 8.0), 1 mM EDTA (pH 8.0), 1× Denhardt’s solution (Eppendorf, Netheler, Germany), 10% dextran sulfate, 10 mM dithiothreitol, 500 μg/ml yeast tRNA, and 7–10 × 10^7^ cpm/ml of ^35^S-uridine 5-triphosphate at 55°C for *Npy*, *Pomc*, *Crf* and *Oxy*. After hybridization, sections were washed three times with 2× saline sodium citrate (SSC)/1 mM DTT at 55°C, treated with 20 μg/ml RNase A (Sigma, St. Louis, MO) at 37°C for 30 min, and then rinsed in 2× SSC/1mM DTT twice at 55°C and washed twice in 0.1× SSC/1mM DTT at 55°C for 15 min. Slides were dehydrated in gradient ethanol, air-dried, and exposed with BMR-2 film (Kodak, Rochester, NY) for 1–3 days to obtain the linear range of the autoradiographs for the semiquantification of mRNA levels. All quantifications were done with films on which the signals were below saturation levels.

### In situ hybridization quantification

Quantitative analysis of the in situ hybridization was performed with National Institutes of Health Scion image software (Bethesda, MD). Autoradiographic images were first scanned on a professional scanner (Epson, Long Beach, CA) and saved to a computer for subsequent analyses with Scion image program using autoradiographic ^14^C microscales (Amersham, Piscataway, NJ) as a standard. Data for each animal represented a mean of the product of hybridization area × density (background density was subtracted) obtained from four to six sections and was normalized to an average of LdLp controls at either PND 23 or PND 90 as 100%.

### Statistical analysis

Data from each group are presented as means ± SEM. Data were analyzed using two-way ANOVA, and followed by pairwise multiple Fisher least significant difference (LSD) comparisons. p < 0.05 was taken to be a statistically significant difference.

## Results

**Fig 1:**
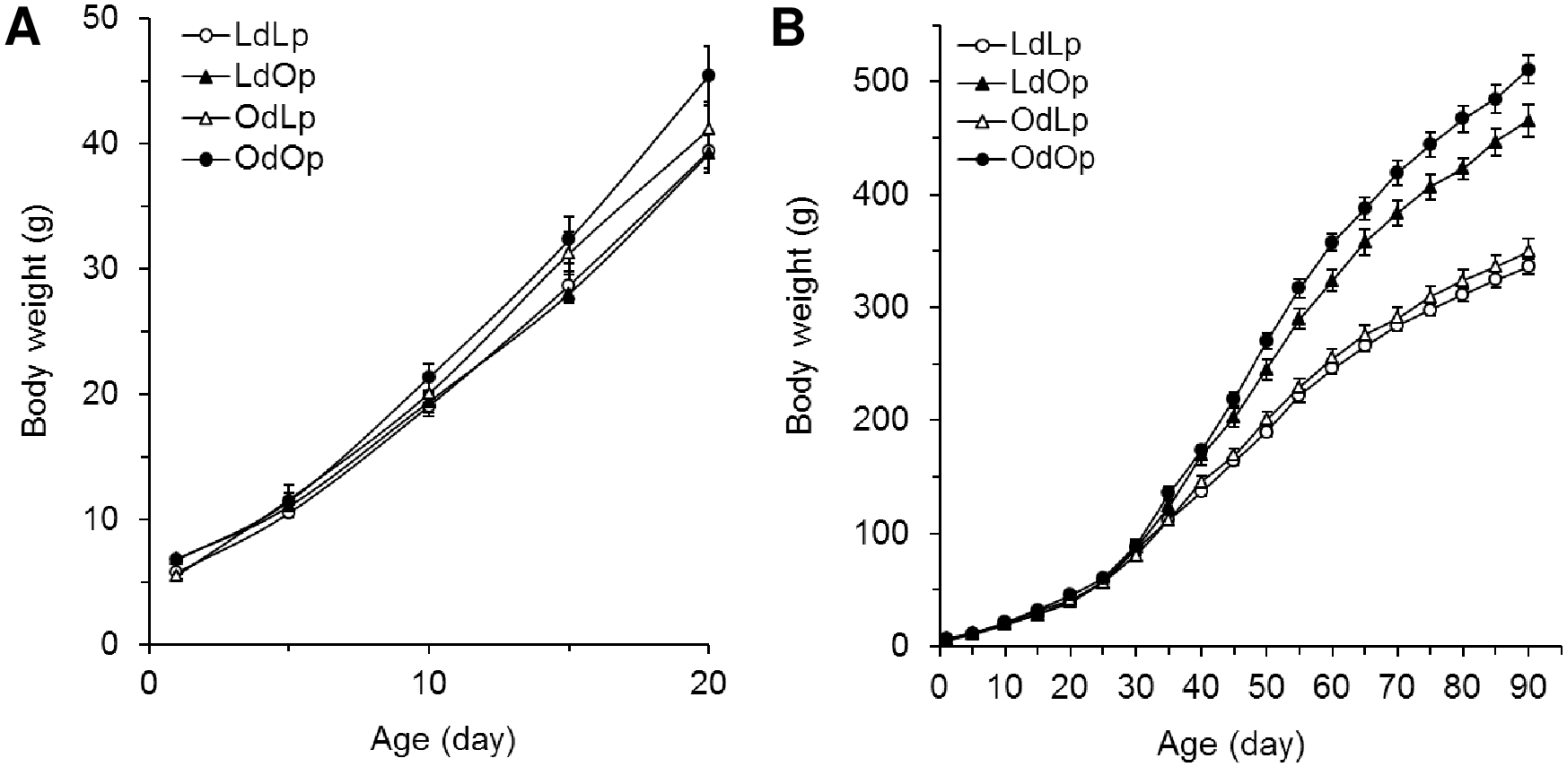
Effect of maternal environment on body weight. (A) Mean body weights during the nursing period and (B) during the 90 day postnatal and post-weaning periods in the 4 groups of offspring (LdLp, LdOp, OdLp, and OdOp–designation of dam (d) and pup (p) genotypes [LETO (L) and OLETF (O) during the sucking period]). OLETF pups gained significantly more weight than did LETO pups. OLETF pups fostered to LETO dams gained less weight than OLETF pups that had been fostered to OLETF dams. Cross fostering had no effect on the weight gain of LETO pups.

Fig 1A shows mean body weight in four groups of LdLp, LdOp, OdLp and OdOp rats prior to weaning. There were significant overall effects of age [F(4, 80) = 3.97, p = .005] and dam environment [F(1, 20) = 4.39, p = .049] but no effect of pup genotype [F(1, 20) = .977, p = .335]. On postnatal days 15 and 20, OdOp weighed more than LdLp pups (p < .05). Fig 1B demonstrates the growth curves out to PND 90. There were significant main effects of dam environment [F(1, 20) = 5.67, p = .03] and pup genotype [F(1, 20) = 117.58, p = .000] as well as age [F(18, 360) = 1.793, p = .025]. OLETF rats continued to gain significantly more body weight than LETO rats. OdOp rats weighed 52% more than LdLp by PND 90. Beginning at postnatal day 50, OLETF rats fostered to LETO (LdOp) dams weighed significantly less than OdOp rats. Body weight did not differ between the two groups of LETO rats fostered to LETO (LdLp) and OLETF dams (OdLp).

**Fig 2:**
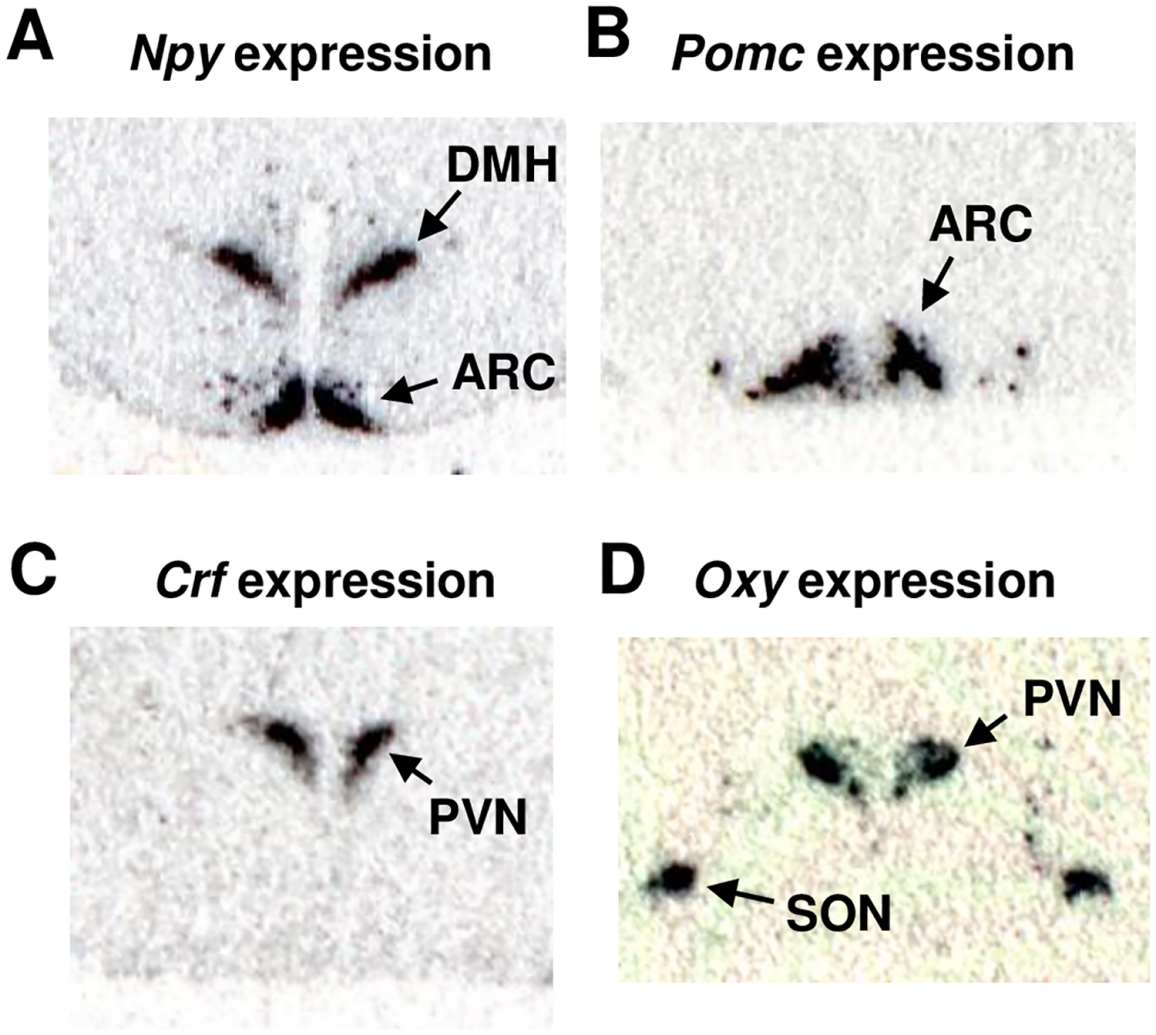
In situ hybridization determinations of hypothalamic gene expression with ^35^S-labeled antisense riboprobes. (A) *Npy* mRNA expression in the dorsomedial hypothalamus (DMH) and the arcuate nucleus (ARC); (B) *Pomc* mRNA expression in the ARC; (C) *Crf* mRNA expression in the paraventricular nucleus (PVN); (D) *Oxy* mRNA expression in the PVN and the supraoptic nucleus (SON).

**Fig 3:**
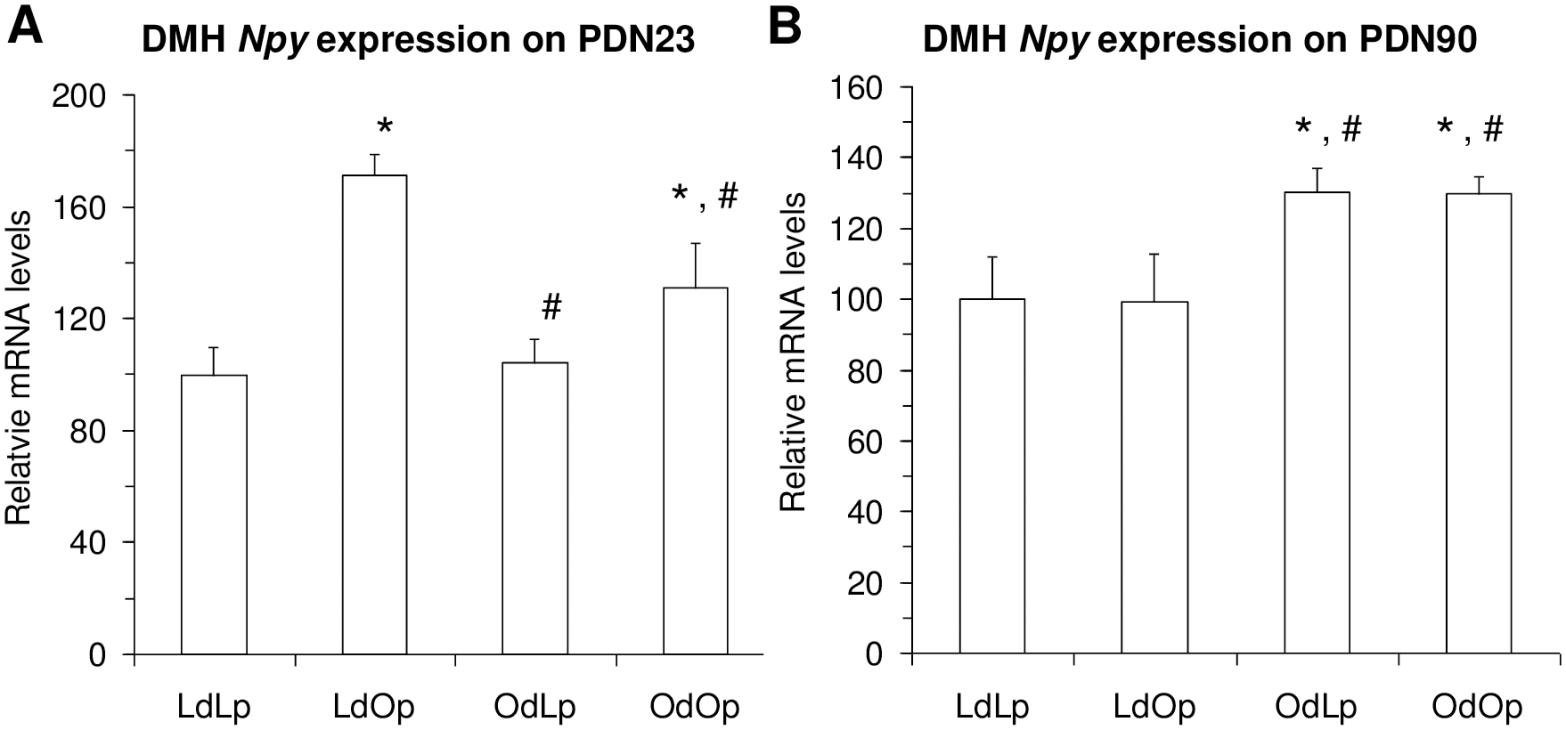
Effects of genotype and maternal environment on DMH *Npy* gene expression at PND 23 (A) and PND 90 (B). At PND 23, two way ANOVA revealed a significant effect of pup offspring genotype and a significant interaction between dam environment and offspring genotype. At PND 90, there was a significant effect of dam environment. *p < .05 vs LdLp and #p < .05 vs LdOp.

Two-way ANOVA revealed a significant effect of pup genotype and a significant interaction between dam environment and pup genotype on *Npy* gene expression in the DMH at PND23 (Figs 2A and 3A). OLETF pups had significantly elevated DMH *Npy* mRNA expression compared to LETO pups and LdOp had significantly higher DMH *Npy* expression than OdOp pups at this age. At PND 90, there was a significant effect of dam environment on DMH *Npy* gene expression. Offspring that were raised by OLETF dams had significantly higher *Npy* gene expression than offspring raised by LETO dams (Fig 3B). There was no effect of offspring genotype in the DMH at PND 90 (Fig 3B).

**Fig 4:**
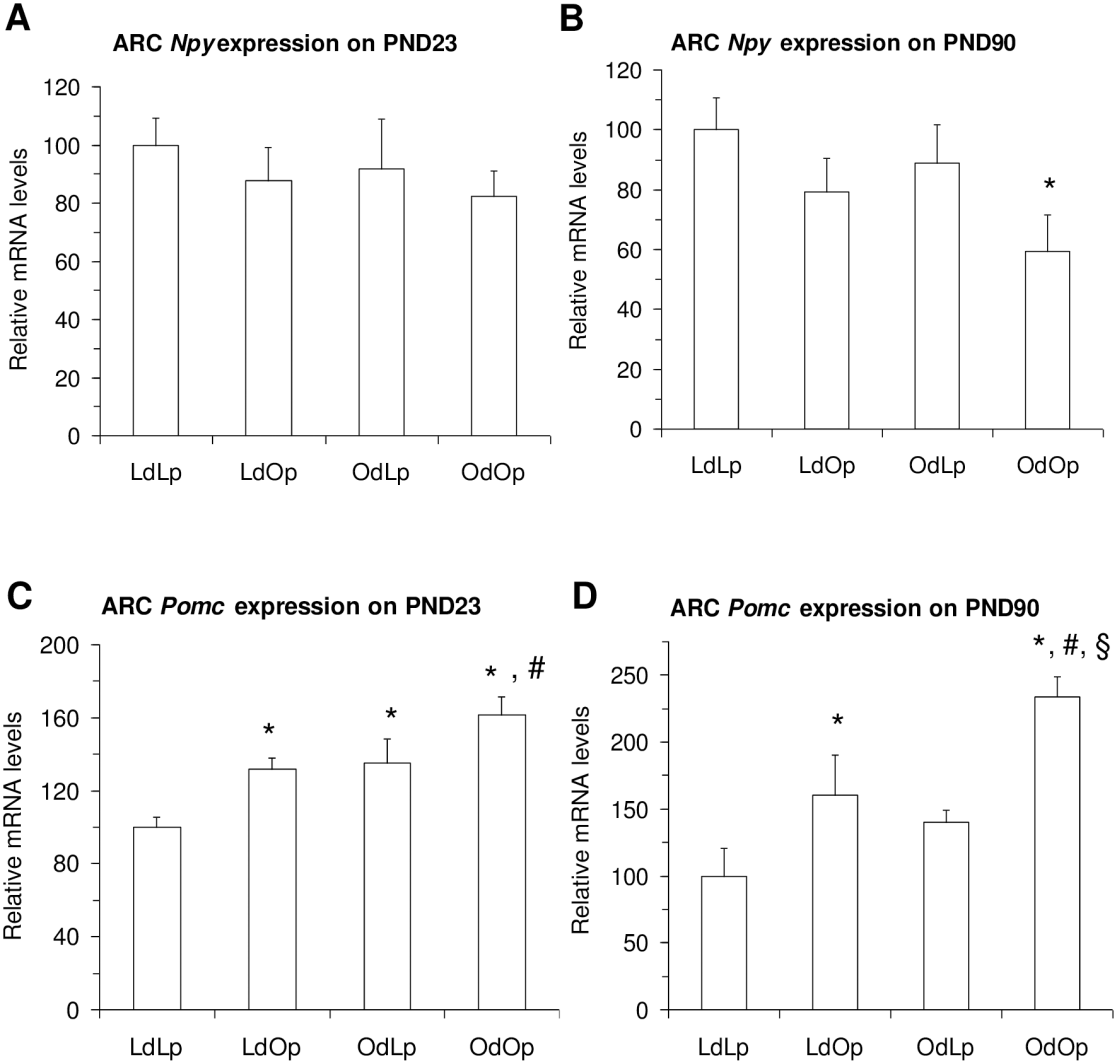
Effects of genotype and maternal environment on ARC *Npy* and *Pomc* gene expression at PND 23 and PND 90. ARC *Npy* gene expression was not affected by either offspring genotype or maternal environment at PND 23 (A), but was significantly affected by offspring genotype at PND 90 (B). There were significant effects of both offspring genotype and maternal environment on ARC *Pomc* gene expression at both PND 23(C) and PND 90 (D). *p < .05 vs. LdLp, #p < .05 vs. LdOp and ^§^p < .05 vs. OdLp.

mRNA expression of orexigenic and anorexigenic peptides in the ARC were differentially affected at PND 23 and PND 90. *Npy* gene expression in the ARC (Fig 2A) was not altered at PND 23 (Fig 4A), but there was a significant effect of pup genotype on PND 90 such that *Npy* gene expression in the OdOp group was significantly reduced relative to levels in LETO offspring (Fig 4B). Determination of *Pomc* gene expression in the ARC (Fig 2B) revealed significant effects of dam environment and pup genotype at both PND 23 (Fig 4C) and 90 (Fig 4D). On PND 23 *Pomc* gene expression in the ARC was elevated in LdOp, OdLp and OdOp relative to that of the LdLp group. In addition, ARC *Pomc* expression was increased in the OdOp group relative to that in the LdOp group. At PND 90, there was a significant effect of genotype in that levels in both groups of OLETF pups were elevated relative to levels in LETO pups (Fig 4D). Additionally, *Pomc* gene expression levels in OdOp pups were elevated relative to levels in LdOp pups (Fig 4D).

**Fig 5:**
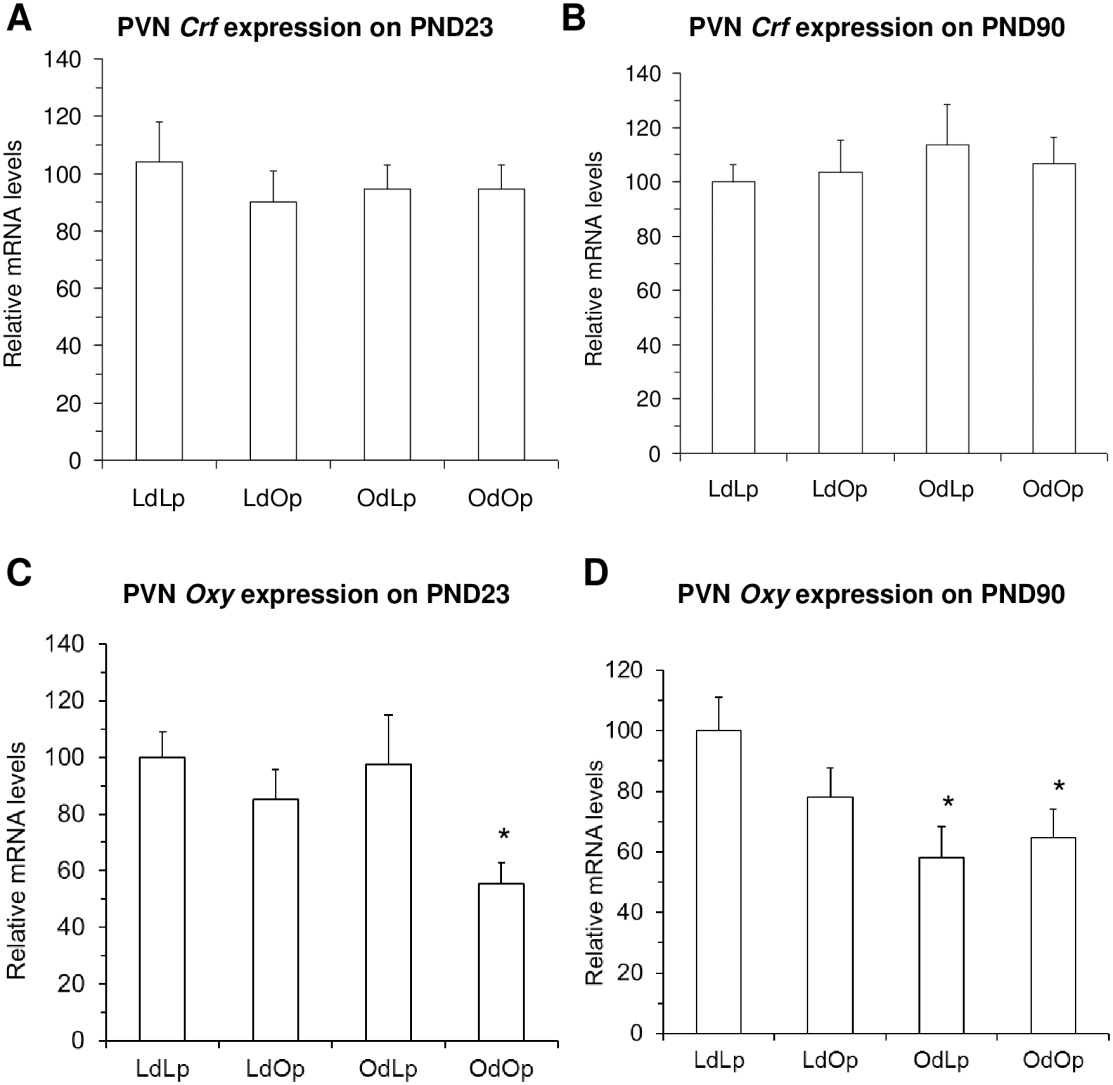
Effects of genotype and maternal environment on PVN *Crf* and O*xt* gene expression at PND 23 and PND 90. There were no effects of offspring genotype or maternal environment on PVN *Cr*f gene expression at either time point (A and B). There was a significant effect of offspring genotype on PVN *Oxy* gene expression at PND 23 (C) and a significant effect of maternal environment on *Oxy* gene expression at PND 90 (D). *p < .05 vs. LdLp.

We examined *Crf* and *Oxy* gene expression in the PVN (Fig 2C and 2D). There were no effects of either pup genotype or maternal environment on *Crf* gene expression (Fig 5A and 5B). In contrast, there was a significant effect of pup genotype on *Oxy* gene expression on PND 23 with lower levels in OLETF pups. This effect was expressed as a significant decrease in the OdOp group relative to levels in LETO pups (Fig 5C). At PND 90, there was a significant effect of maternal environment with elevated levels of *Oxy* gene expression in offspring raised by OLETF dams (Fig 5D).

## Discussion

This study was designed to clarify the role of the postnatal environment in modulating the expression of hypothalamic genes involved in energy balance in a genetic obesity model: the OLETF rat. Prior work by our group has demonstrated that the early postnatal environment affects the development of obesity in male OLETF rats. Male OLETF rats raised by LETO dams have attenuated obesity following weaning [19] and the present results replicate that finding. The data further demonstrate effects of maternal environment on patterns of hypothalamic gene expression.

DMH NPY has been implicated in the hyperphagia and obesity of OLETF rats. Prior work has demonstrated that DMH *Npy* gene expression is significantly elevated prior to obesity development in young OLETF rats and is highly elevated in adult OLETF rats pair fed to amounts of food consumed by control LETO rats [14,15]. Furthermore, knockdown of NPY in the DMH normalizes food intake and body weight in the OLETF rats. The current data demonstrate that the maternal environment modulates DMH *Npy* expression. As previously demonstrated, DMH *Npy* expression is significantly elevated in the OLETF rats reared by OLETF dams at the time of weaning. Interestingly, DMH *Npy* gene expression is not only elevated in the OLETF pups reared by control LETO dams but is higher than the level found in the OdOp rats at PND 23. We have shown previously that the combination of OLETF dams and OLETF pups provide a richer nursing environment, resulting in shorter latencies to initiate nursing, extended nursing time and greater intake [19]. Even though there was not a significant weight difference between OdLp and OdOp pups at weaning, the OdLp group was slightly heavier than the LdLp group and this may reflect the difference in nursing interactions. The decrease in nursing interaction in LdOp pups may account for the further elevation in DMH *Npy* expression at weaning similar to how food restriction elevates DMH *Npy* gene expression in Sprague Dawley rats [21].

There was a significant effect of maternal environment on DMH *Npy* expression at PND 90. Both LETO and OLETF rats raised by OLETF dams had elevated expression of *Npy* in the DMH relative to levels from rats raised by LETO dams. Prior results have produced somewhat conflicting data on the DMH *Npy* expression in adult ad lib fed OLETF rats. Initial data found a nonsignificant elevation [14] while a subsequent study in a control group for a running wheel experiment found a significant 33% increase in adult sedentary OLETF rats compared with LETO controls [22]. The present results are comparable to these latter data. In addition, we observed that *Npy* mRNA expression in the DMH of LdOp rats was reduced on PND 90. This change may contribute to their reduction of body weight relative to OdOp rats. We do not have a good explanation for the elevation in the LETO rats reared by OLETF dams.

In contrast to DMH *Npy* gene expression, ARC *Npy* expression was not affected by either the pup genotype or the maternal environment at the time of weaning. ARC *Npy* expression was decreased in OdOp rats at PND 90 as we have previously shown [14]. This was normalized in the OLETF rats reared by LETO dams. This normalization may reflect the reduced body weight in this group. We have previously hypothesized that the decrease in ARC *Npy* expression in adult OLETF rats may reflect a regulatory response to their obesity [14]. The reduced body weight in LdOp rats may provide an explanation for the normalization of their Arc *Npy* expression. There were effects of both pup genotype and maternal environment on ARC *Pomc* gene expression at weaning. *Pomc* gene expression was elevated by both the maternal environment and by the pup genotype with an apparent additive effect resulting in the level in the OdOp group being higher than the others. Others have previously demonstrated a normal response to leptin in OLETF rats [23] and thus the elevated levels of *Pomc* gene expression at this age may reflect elevations in plasma leptin as has been demonstrated in LETO pups raised by OLETF dams [19]. However, levels of leptin were not elevated in OLETF pups raised by either OLETF or LETO dams at weaning despite elevated levels of body fat [19]. At postnatal day 90, the levels of *Pomc* gene expression appear to reflect a response to the body weights with elevations in both OLETF groups but a modulation by the maternal phenotype consistent with the body weight results.

There were no effects of either genotype or maternal environment on PVN *Crf* gene expression. In contrast, PVN *Oxy* expression was reduced in OdOp rats at PND23 and in both OLETF and LETO rats reared by OLETF dams at day 90. This pattern is the opposite of what we found for DMH *Npy* gene expression raising the possibility that DMH NPY down-regulates oxytocin expression in the PVN. Central oxytocin administration has been demonstrated to inhibit food intake [24] and PVN oxytocin has previously been postulated to mediate the effects of leptin on meal size [25]. Thus, the signaling pathway of DMH NPY regulation of PVN oxytocin in modulating food intake merits further investigation.

## Conclusions

Overall, these data further demonstrate effects of maternal environment on the eventual metabolic phenotype. The results on body weight replicate our previous finding in that the LETO maternal environment modulates the body weight trajectory of OLETF pups. Prior work has also demonstrated that the maternal environment modulates overall food intake both in independent ingestion tests in pups as well as subsequent to weaning [19]. While both OLETF and LETO dams modulated behavior during nursing and resulted in alterations in body fat and leptin levels, only the effects of the LETO dam on modulating OLETF offspring were evident in long term body weight data. However, the current data demonstrate lasting effects of both maternal environments on patterns of hypothalamic gene expression.

## Supporting information

S1 Table Statistical information for Fig 1. (DOCX)

S2 Table Statistical information for Fig 3. (DOCX)

S3 Table Statistical information for Fig 4. (DOCX)

S4 Table Statistical information for Fig 5. (DOCX)
