# Supplementary material for: Effects of early postnatal environment on hypothalamic gene expression in OLETF rats
Source: PLoS One. 2017 Jun 2;12(6):e0178428. doi: 10.1371/journal.pone.0178428 (PMC5456065; doi:10.1371/journal.pone.0178428)
Supplement: S1 Table — (DOCX) [file pone.0178428.s001.docx]

**S1 Table. Effect of maternal environment on body weight.**

| Repeated Measures Analysis of Variance over 90 days Sigma-restricted parameterization  Effective hypothesis decomposition | | | | | |
| --- | --- | --- | --- | --- | --- |
|  | **SS** | **Degr. of** | **MS** | **F** | **p** |
| **Intercept** | 17570457 | 1 | 17570457 | 5608.250 | 0.000000 |
| **Dam** | 17751 | 1 | 17751 | 5.666 | 0.027359 |
| **pup** | 368379 | 1 | 368379 | 117.581 | 0.000000 |
| **Dam*pup** | 5268 | 1 | 5268 | 1.681 | 0.209505 |
| **Error** | 62659 | 20 | 3133 |  |  |
| **R1** | 9511620 | 18 | 528423 | 4313.384 | 0.000000 |
| **R1*Dam** | 12622 | 18 | 701 | 5.724 | 0.000000 |
| **R1*pup** | 322976 | 18 | 17943 | 146.465 | 0.000000 |
| **R1*Dam*pup** | 3955 | 18 | 220 | 1.793 | 0.024558 |
| **Error** | 44103 | 360 | 123 |  |  |
