# Supplementary material for: Effects of early postnatal environment on hypothalamic gene expression in OLETF rats
Source: PLoS One. 2017 Jun 2;12(6):e0178428. doi: 10.1371/journal.pone.0178428 (PMC5456065; doi:10.1371/journal.pone.0178428)
Supplement: S2 Table — (DOCX) [file pone.0178428.s002.docx]

**S2 Table. Effects of genotype and maternal environment on DMH *Npy* gene expression at PND 23 and PND 90**.

| Univariate Tests of Significance at PND 23 Sigma-restricted parameterization  Effective hypothesis decomposition | | | | | |
| --- | --- | --- | --- | --- | --- |
|  | **SS** | **Degr. of** | **MS** | **F** | **p** |
| **Intercept** | 384863.5 | 1 | 384863.5 | 600.8625 | 0.000000 |
| **Dam** | 1959.2 | 1 | 1959.2 | 3.0587 | 0.095637 |
| **Pup** | 14519.8 | 1 | 14519.8 | 22.6687 | 0.000119 |
| **Dam*Pup** | 2942.0 | 1 | 2942.0 | 4.5932 | 0.044580 |
| **Error** | 12810.4 | 20 | 640.5 |  |  |

| Univariate Tests of Significance at PND 90 Sigma-restricted parameterization  Effective hypothesis decomposition | | | | | |
| --- | --- | --- | --- | --- | --- |
|  | **SS** | **Degr. of** | **MS** | **F** | **p** |
| **Intercept** | 316648.5 | 1 | 316648.5 | 610.4253 | 0.000000 |
| **Dam** | 5557.0 | 1 | 5557.0 | 10.7126 | 0.003805 |
| **Pup** | 1.4 | 1 | 1.4 | 0.0027 | 0.959348 |
| **Dam*Pup** | 0.3 | 1 | 0.3 | 0.0006 | 0.980896 |
| **Error** | 10374.7 | 20 | 518.7 |  |  |
