# Supplementary material for: Effects of early postnatal environment on hypothalamic gene expression in OLETF rats
Source: PLoS One. 2017 Jun 2;12(6):e0178428. doi: 10.1371/journal.pone.0178428 (PMC5456065; doi:10.1371/journal.pone.0178428)
Supplement: S3 Table — (DOCX) [file pone.0178428.s003.docx]

**S3 Table. Effects of genotype and maternal environment on ARC *Npy* and *Pomc* gene expression at PND 23 and PND 90**.

| Univariate Tests of Significance for ARC NPY at PND 23 Sigma-restricted parameterization  Effective hypothesis decomposition | | | | | |
| --- | --- | --- | --- | --- | --- |
|  | **SS** | **Degr. of** | **MS** | **F** | **p** |
| **Intercept** | 196251.8 | 1 | 196251.8 | 220.5745 | 0.000000 |
| **Dam** | 734.1 | 1 | 734.1 | 0.8251 | 0.374508 |
| **Pup** | 10.9 | 1 | 10.9 | 0.0122 | 0.913112 |
| **Dam*Pup** | 272.4 | 1 | 272.4 | 0.3061 | 0.586190 |
| **Error** | 17794.6 | 20 | 889.7 |  |  |

| Univariate Tests of Significance for ARC NPY at PND 90 Sigma-restricted parameterization  Effective hypothesis decomposition | | | | | |
| --- | --- | --- | --- | --- | --- |
|  | **SS** | **Degr. of** | **MS** | **F** | **p** |
| **Intercept** | 160728.8 | 1 | 160728.8 | 199.8406 | 0.000000 |
| **Dam** | 1432.2 | 1 | 1432.2 | 1.7807 | 0.197056 |
| **Pup** | 3807.8 | 1 | 3807.8 | 4.7344 | 0.041726 |
| **Dam*Pup** | 111.6 | 1 | 111.6 | 0.1388 | 0.713438 |
| **Error** | 16085.7 | 20 | 804.3 |  |  |

| Univariate Tests of Significance for ARC POMC at PND 23 Sigma-restricted parameterization  Effective hypothesis decomposition | | | | | |
| --- | --- | --- | --- | --- | --- |
|  | **SS** | **Degr. of** | **MS** | **F** | **p** |
| **Intercept** | 381478.3 | 1 | 381478.3 | 799.1004 | 0.000000 |
| **Dam** | 5791.9 | 1 | 5791.9 | 12.1325 | 0.002654 |
| **Pup** | 4570.8 | 1 | 4570.8 | 9.5746 | 0.006257 |
| **Dam*Pup** | 31.9 | 1 | 31.9 | 0.0669 | 0.798807 |
| **Error** | 8592.9 | 18 | 477.4 |  |  |

| Univariate Tests of Significance for ARC POMC at PND 90 Sigma-restricted parameterization  Effective hypothesis decomposition | | | | | |
| --- | --- | --- | --- | --- | --- |
|  | **SS** | **Degr. of** | **MS** | **F** | **p** |
| **Intercept** | 604139.1 | 1 | 604139.1 | 286.9326 | 0.000000 |
| **Dam** | 19443.7 | 1 | 19443.7 | 9.2347 | 0.006482 |
| **Pup** | 35689.1 | 1 | 35689.1 | 16.9503 | 0.000535 |
| **Dam*Pup** | 1680.2 | 1 | 1680.2 | 0.7980 | 0.382315 |
| **Error** | 42110.2 | 20 | 2105.5 |  |  |
