# Supplementary material for: Effects of early postnatal environment on hypothalamic gene expression in OLETF rats
Source: PLoS One. 2017 Jun 2;12(6):e0178428. doi: 10.1371/journal.pone.0178428 (PMC5456065; doi:10.1371/journal.pone.0178428)
Supplement: S4 Table — (DOCX) [file pone.0178428.s004.docx]

**S4 Table. Effects of genotype and maternal environment on PVN *Crf* and O*xt* gene expression at PND 23 and PND 90**.

| Univariate Tests of Significance for PVN CRF at PND 23 Sigma-restricted parameterization  Effective hypothesis decomposition | | | | | |
| --- | --- | --- | --- | --- | --- |
|  | **SS** | **Degr. of** | **MS** | **F** | **p** |
| **Intercept** | 220551.9 | 1 | 220551.9 | 329.1748 | 0.000000 |
| **Dam** | 34.0 | 1 | 34.0 | 0.0508 | 0.824038 |
| **Pup** | 278.6 | 1 | 278.6 | 0.4158 | 0.526381 |
| **Dam*Pup** | 278.6 | 1 | 278.6 | 0.4158 | 0.526381 |
| **Error** | 13400.3 | 20 | 670.0 |  |  |

| Univariate Tests of Significance for PVN CRF at PND 90 Sigma-restricted parameterization  Effective hypothesis decomposition | | | | | |
| --- | --- | --- | --- | --- | --- |
|  | **SS** | **Degr. of** | **MS** | **F** | **p** |
| **Intercept** | 396105.7 | 1 | 396105.7 | 534.1942 | 0.000000 |
| **Dam** | 623.7 | 1 | 623.7 | 0.8411 | 0.370018 |
| **Pup** | 29.6 | 1 | 29.6 | 0.0399 | 0.843622 |
| **Dam*Pup** | 253.2 | 1 | 253.2 | 0.3415 | 0.565516 |
| **Error** | 14830.0 | 20 | 741.5 |  |  |

| Univariate Tests of Significance for PVN Oxy at PND 23 Sigma-restricted parameterization  Effective hypothesis decomposition | | | | | |
| --- | --- | --- | --- | --- | --- |
|  | **SS** | **Degr. of** | **MS** | **F** | **p** |
| **Intercept** | 140084.2 | 1 | 140084.2 | 209.5774 | 0.000000 |
| **Dam** | 1283.8 | 1 | 1283.8 | 1.9206 | 0.184800 |
| **Pup** | 3958.0 | 1 | 3958.0 | 5.9215 | 0.027055 |
| **Dam*Pup** | 923.8 | 1 | 923.8 | 1.3821 | 0.256942 |
| **Error** | 10694.6 | 16 | 668.4 |  |  |

| Univariate Tests of Significance for PVN oxy at PND 90 Sigma-restricted parameterization  Effective hypothesis decomposition | | | | | |
| --- | --- | --- | --- | --- | --- |
|  | **SS** | **Degr. of** | **MS** | **F** | **p** |
| **Intercept** | 135794.4 | 1 | 135794.4 | 217.8840 | 0.000000 |
| **Dam** | 4547.9 | 1 | 4547.9 | 7.2972 | 0.013738 |
| **Pup** | 364.4 | 1 | 364.4 | 0.5847 | 0.453416 |
| **Dam*Pup** | 1215.8 | 1 | 1215.8 | 1.9507 | 0.177819 |
| **Error** | 12464.8 | 20 | 623.2 |  |  |
